# A Strategy for Preparative Separation of 10 Lignans from *Justicia procumbens* L. by High-Speed Counter-Current Chromatography

**DOI:** 10.3390/molecules22122024

**Published:** 2017-11-23

**Authors:** Jiaojiao Jiang, Hongjing Dong, Tao Wang, Ruixuan Zhao, Yan Mu, Yanling Geng, Zhenjia Zheng, Xiao Wang

**Affiliations:** 1 Key Laboratory of TCM Quality Control Technology, Shandong Analysis and Test Center, Qilu University of Technology (Shandong Academy of Sciences), Jinan 250014, China; 13165144592@163.com (J.J.); donghongjing_2006@163.com (H.D.); foxfriendship@163.com (T.W.); my3634@163.com (Y.M.); gengyanling@126.com (Y.G.); 2 College of Pharmacy, Shandong University of Traditional Chinese Medicine, Jinan 250355, China; 3 College of Food Science and Engineering, Shandong Agricultural University, Taian 271018, China; zhaoruixuan2016@126.com

**Keywords:** *Justicia procumbens* L., lignans and their glycosides, preparative separation, high-speed counter-current chromatography

## Abstract

Ten compounds, including three lignan glycosides and seven lignans, were purified from *Justicia procumbens* L. in 8 h using an efficient strategy based on high-speed counter-current chromatography (HSCCC). The two-phase solvent system composed of petroleum–ethyl acetate–methanol–H_2_O (1:0.7:1:0.7, *v*/*v*) was firstly employed to separate the crude extract (320 mg), from which 19.3 mg of justicidin B (**f**), 10.8 mg of justicidin A (**g**), 13.9 mg of 6′-hydroxyjusticidin C (**h**), 7.7 mg of justicidin E (**i**), 6.3 mg of lignan J_1_ (**j**) were obtained with 91.3 mg of enriched mixture of compounds **a**–**e**. The enriched mixture (91.3 mg) was further separated using the solvent system consisting of petroleum–ethyl acetate–methanol–H_2_O (3:3.8:3:3.8, *v*/*v*), yielding 12.1 mg of procumbenoside E (**a**); 7.6 mg of diphyllin-1-*O*-β-d-apiofuranoside (**b**); 7.4 mg of diphyllin (**c**); 8.3 mg of 6′-hydroxy justicidin B (**d**); and 7.9 mg of diphyllin acetyl apioside (**e**). The purities of the 10 components were all above 94%, and their structures were identified by NMR and ESI-MS spectra. The results demonstrated that the strategy based on HSCCC for the separation of lignans and their glycosides was efficient and rapid.

## 1. Introduction

*Justicia procumbens* L. (Chinese name Juechuang) is one of the famous traditional Chinese herbal medicine, which stemmed from the dried whole plant of the genus Acanthaceae [1]. It has detoxification and diuretic swelling effect for the treatment of fever, cough, sore throat, cirrhosis of the ascites [2,3]. Previous phytochemical studies indicated that *J. procumbens* contains various ingredients, including lignans, lignan glycosides, alkaloids, flavonoids, triterpenes, steroids, etc. [4,5,6]. Modern pharmacological investigations indicated that lignans and their glycosides are the main chemical components in *J. procumbens*, which exhibit various pharmacological activities, including antitumor, antivirus, antihepatitis, inhibition of platelet aggregation, and cytotoxicity [7,8,9,10]. In order to better understand the biochemical properties of lignans and their glycosides from *J. procumbens* and to ascertain their clinical applications, it is essential to develop an efficient method for the preparative separation of lignans and their glycosides.

**Figure 1:**
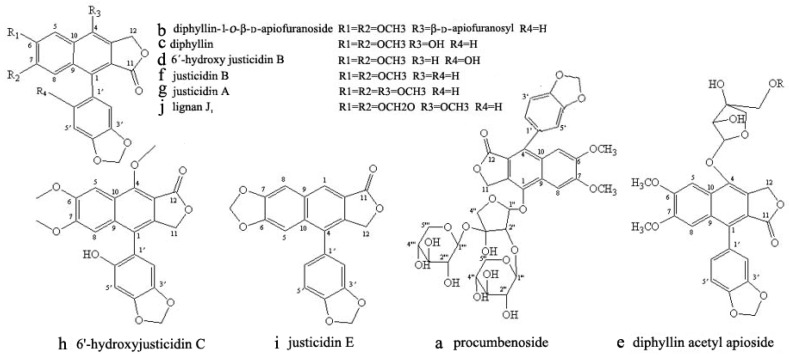
Chemical structures of 10 lignans from *Justicia procumbens.*

Solid-phase chromatography was often used to separate lignans and their glycosides, which is time-consuming and laborious [5,9,11]. High-speed counter-current chromatography (HSCCC) used liquid as a carrier, which effectively avoided the solid carrier adsorption, sample loss, etc. [12,13,14,15,16]. With its high recovery rates and excellent repeatability, the application of HSCCC in preparation separation of bioactive ingredients from natural products is steadily increasing [17,18,19,20,21,22,23]. HSCCC has been reported by Zhou et al. for isolating the lignans [24], however, only four lignans—including justicidin B, justicidin A, 6′-hydroxyjusticidin C, and lignan J_1_—were obtained, and the main components of lignans glycosides were not concerned. In this paper, 10 compounds including 7 lignans and 3 lignan glycosides (Figure 1), were obtained from *J. procumbens*, and the total yield of 10 compounds was 31.7%. 

## 2. Results and Discussion

### 2.1. Selection of the Two-Phase Solvent System

Selection of solvent system is the key step for the HSCCC separation, usually by measuring the *K*_D_ value of target compounds to determine the suitable two-phase solvent system. For the isolation of HSCCC, the optimum range of the *K*_D_ value of target compounds in two-phase solvent system is 0.5–2 [25,26,27]. According to the chemical properties of lignans, in this paper, the *K*_D_ values of 10 components in *J. procumbens* were measured by HPLC in a series of different solvent systems compose of petroleum (Pet)–ethyl acetate (EtOAc)–methanol (MeOH)–H_2_O at various volume ratios.

**Figure 2:**
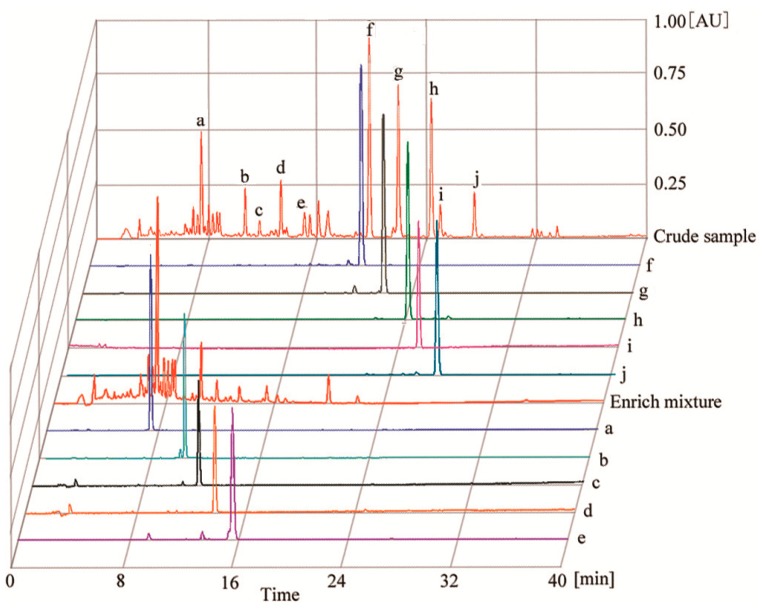
HPLC chromatograms of crude sample, enriched mixture (compounds **a**–**e**) and purified lignans (compounds **a**–**j**) from *J. procumbens*. Experimental conditions: RP-C_18_ column (5 µm, 4.6 mm × 250 mm; Waters Technologies, USA); mobile phase: acetonitrile (A) 0.1% TFA water solution (B) (0–10 min, 30–45% A; 10–25 min, 45–60% A; 25–40 min, 60–100% A); RP-C_18_ column temperature: 25 °C, UV monitor wavelength: 254 nm; flow-rate: 1 mL·min^−1^; injection volume: 20 µL. **a**. procumbenoside E; **b**. diphyllin-1-*O*-β-d-apiofuranoside; **c**. diphyllin; **d**. 6′-hydroxy justicidin B; **e**. diphyllin acetyl apioside; **f**. justicidin B; **g**. justicidin A; **h**. 6′-hydroxyjusticidin C; **i**. justicidin E; **j**. lignan J_1_.

**Figure 3:**
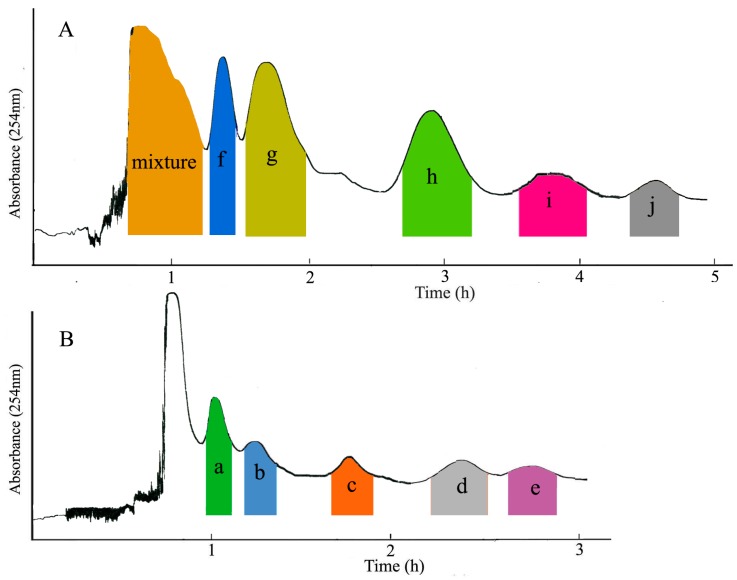
HSCCC chromatograms of crude sample and enriched mixture (compounds **a**–**e**). Experimental conditions of crude sample (**A**): solvent system: Pet–EtOAc–MeOH–H_2_O (1:0.7:1:0.7, *v*/*v*); sample size: 320 mg; retention of the organic phase: 62.5%. Conditions of the enriched mixture (**B**): solvent system: Pet–EtOAc–MeOH–H_2_O (3:3.8:3:3.8, *v*/*v*); sample size: 91.3 mg. retention of the organic phase: 53.1%. In both separations: flow-rate: 3.0 mL∙min^−1^; rotational speed: 810 rpm; UV monitor: 254 nm.

The *K*_D_ value of the 10 target compounds were summarized in Table 1. As shown in Table 1, there are no satisfactory *K*_D_ values for the 10 components in a single two-phase solvent system, and it is impossible that the 10 components were isolated by a single-step CCC separation. However, Pet–EtOAc–MeOH–H_2_O (1:0.7:1:0.7, *v*/*v*) provided suitable *K*_D_ values for compounds **f**–**j** (Figure 2), when this solvent system was applied in CCC apparatus at the conventional flow-rate of 2.0 mL·min^−1^, compounds **f**–**j** could be purified with good resolution and compounds **a**–**e** (Figure 2) were eluted together as an enriched mixture near the solvent front. The whole separation process took more than 6 h, but it would be shortened by increasing the flow rate. However, the flow rate reached up to 4 mL·min^−1^, loss of the stationary phase caused the low resolution between compounds **f** and **g**. However, when the flow rate was 3.0 mL·min^−1^, satisfactory isolation with high efficiency and resolutions was acquired in 5 h (Figure 3A).

Based on the *K*_D_ values of compounds **a**–**e** in Pet–EtOAc–MeOH–H_2_O (1:0.7:1:0.7, *v*/*v*), the solvent system composed of Pet–EtOAc–MeOH–H_2_O may be valid for the separation of the enriched mixture. In further research, the ratio of EtOAc and H_2_O were increased to reduce the elution ability of the mobile phase. The *K*_D_ values of compounds **a**–**e** in a series of solvent system composed of Pet–EtOAc–MeOH–H_2_O at different ratios were evaluated (Table 1), and Pet–EtOAc–MeOH–H_2_O (3:3.8:3:3.8, *v*/*v*) provide suitable *K*_D_ values for compounds **a**–**e**, and when this solvent system was used for separation of the enriched mixture at the flow rate of 3.0 mL·min^−1^, five irregular peaks were presented in 3 h, which indicating the successful separation of compounds **a**–**e** (Figure 3B). 

Therefore, the solvent system composed of Pet–EtOAc–MeOH–H_2_O (1:0.7:1:0.7, *v*/*v*) was applied to purify the crude sample, and the HSCCC absorption curve was presented in Figure 3A. Subsequent HPLC analysis indicated that a mixture, justicidin B (peak **f**), justicidin A (peak **g**), 6′-hydroxyjusticidin C (peak **h**), justicidin E (peak **i**), and lignan J_1_ (peak **j**) were obtained. For the further separation, the enriched mixture was purified by HSCCC using Pet–EtOAc–MeOH–H_2_O (3:3.8:3:3.8, *v*/*v*) as the solvent system. Figure 3B shows that five major peaks were gained. Five HSCCC peaks were analyzed by HPLC showed that the five peaks were procumbenoside E (peak **a**), diphyllin-1-*O*-β-d-apiofuranoside (peak **b**), diphyllin (peak **c**), 6′-hydroxy justicidin B (peak **d**), and diphyllin acetyl apioside (peak **e**), respectively.

### 2.2. *HPLC* Analysis of *HSCCC* Peak Fractions

The crude sample, the enriched mixture and each peak collection from HSCCC were all analyzed by HPLC with a gradient elution mode. After analyzed by HPLC in sequence based on the peak area normalization method at the optimized detection wavelength of 260 nm, the purities of 10 compounds were 98.7% (**a**), 96.2% (**b**), 95.6% (**c**), 95.0% (**d**), 94.1% (**e**), 97.6% (**f**), 95.4% (**j**), 97.3% (**h**), 98.1% (**i**) and 97.3% (**j**), respectively, as shown in Figure 2. 

## 3. Experimental

### 3.1. Material and Reagents

Dried whole plant of *J. procumbens* was purchased from Bozhou drug market (Bozhou, Anhui province, China), and identified by Professor Jia Li (Shandong University of Traditional Chinese Medicine, Jinan, China).

Petroleum ether (Pet) (60–90 °C), ethyl acetate (EtAc), methanol (MeOH), and chloroform were all of analytical grade and purchased from Tianjin Chemical Reagent Factory (Tianjin, China). Acetonitrile and trifluoroacetic acid (TFA) employed for HPLC analysis were purchased from Shanghai Baili Bio-technology Co., Ltd. (Shanghai, China). The distilled water (Beijing Boao Biological Co., Ltd., Beijing, China) was used for all pure and solutions.

### 3.2. Apparatus

HSCCC separation was performed by TBE-300C (Tauto Biotech, Shanghai, China), which was armed with three multilayer coils connected in series (total volume: 320 mL, the diameter: 2.6 mm) with a sample loop (20 mL). Rotation speed of the apparatus can be controlled up to 1000 rpm with a speed controller. During the experiment, the temperature of the HSCCC equipment is controlled at 25 °C by the DCW-0506 low constant temp monitor (Tauto Biotech, Shanghai, China). The pumping of the two-phase system was performed by the BT100-2J constant flow pump (Longer Pump Co., Ltd., Baoding, China). Spectra were produced from an 8823B-UV detector (Asahi Technology Co., Ltd., Hangzhou, China) at 254 nm. The description of the CCC diagram was implemented by a model MTK 1000 recorder (Hangzhou Mico Technology Co., Ltd., Hangzhou, China). 

The sample was analyzed by HPLC Agilent 1120 (Agilent Technologies, Santa Clara, CA, USA) equipped with a G1315C diode array detector (DAD) system, an Agilent 1120 binary-solvent delivery system, an automatic sample injection, and an Empower 3 work-station.

### 3.3. Pre-Processing of Crude Sample

The dried whole plant (5 kg) of *J. procumbens* was fully crushed into powder and extracted three times (3 h, 2 h, 2 h) with 95% ethanol under reflux. The extract was filtered and concentrated by rotary evaporation at 55 °C to remove ethanol completely Then, equal amount of ethyl acetate (1 L) was applied to extract the concentrate three times, yielding 87.5 g of crude sample which was stored in refrigerator at 4 °C for further HSCCC purification.

### 3.4. Measurement of the Partition Coefficients (K_D_)

Pet-EtAc-MeOH-H_2_O as the optimized solvent system was used to measure the partition coefficients (*K*_D_) value of the target compounds by HPLC as follows: Approximately 2 mg of crude sample was placed in a test tube, to which 1.5 mL equilibrated two-phase solvent system were added. The sample was fully dissolved in the solvent system by violently shaking the tube. Then, equal amounts of the organic and aqueous phases were separately analyzed by HPLC. The *K*_D_ values of target compounds are defined as

*K*_D_ = *A_s_*/*A_m_*

*A_s_*: The HPLC peak area of the target components in the stationary phase; *A_m_*: The HPLC peak area of target components in the mobile phase.

### 3.5. Preparation of Solvent Systems and Sample Solutions

Two-phase solvent systems composed of Pet–EtOAc–MeOH–H_2_O (1:0.7:1:0.7, *v*/*v*) and Pet–EtOAc–MeOH–H_2_O (3:3.8:3:3.8, *v*/*v*) were applied in the HSCCC separation. Each solvent mixture was thoroughly equilibrated in a separatory funnel for 2 h at room temperature. Shortly before use, each solvent system was separated into two phases and each phase was degassed separately.

The sample solutions were prepared by dissolving 320 mg of the crude sample in 5 mL of each phase (total of 10 mL) used for separation.

### 3.6. Separation Procedure

In each separation process, the upper phase was firstly pumped into the three multilayer-coil combined in series at 40 mL·min^−1^. Then, when the CCC instrument rotational speed reaches 810 rpm in the positive direction, the mobile phase was introduced into the column with 3.0 mL·min^−1^. When the hydrodynamic equilibrium between the two phases was reached, the sample solution was put into the sample loop, while the UV detector (254 nm) monitored the effluent continuously. The peaks were gathered manually in accordance with the UV absorption curve and then subjected to HPLC analysis. Finally, measure the ratio of the organic phase to the whole volume in the multilayer-coiled columns to obtain the retention of the organic phase. 

### 3.7. *HPLC* Analysis of *CCC* Separation Products

The peak fraction from CCC separation, crude sample, and the enriched mixture were analyzed by HPLC equipped with a G1315C diode array detector (DAD) system. A RP-C_18_ column (5 µm, 4.6 mm × 250 mm; Waters Technologies, Milford, MA, USA) at 25 °C was applied for all the analyses. The flow-rate was get to 1 mL·min^−1^, and the monitor wavelength was 254 nm. Using the mobile phase composed of A (acetonitrile) and B (water including of 0.1% TFA), and the gradient elution was carried out as follows: 0 min, 30% A; 0–10 min, 30–45% A; 10–25 min, 45–60% A; 25–40 min, 60–100% A.

### 3.8. Identification of *CCC* Fractions

The pure compounds obtained by the CCC separation were analyzed by ^1^H-NMR and ^13^C-NMR with a Varian-600 spectrometer (Varian, Palo Alto, CA, USA)and detected the molecular weight by an Agilent 1100/MS-G1946 (Agilent, Santa Clara, CA, USA) mass selective detector. The detail data of each component was as follows:

Compound **a** (peak **a** in Figure 3B): Positive ESI-MS (*m*/*z*): 777.0 [M + H]^+^. ^1^H-NMR (DMSO-*d*_6_, 400 MHz) δ: 6. 94 (1H, d, *J* = 1.1 Hz, H-2′), 7.03 (1H, d, *J* = 7.9 Hz, H-5′), 6.77 (1H, dd, *J* = 7.9, 1.1 Hz, H-6′), 7.00 (1H, s, H-5), 7.64 (1H, s, H-8), 3.98 (3H, s, OCH_3_-6), 3.64 (3H, s, OCH_3_-7), 6.13 (2H, s, 3′-OCH_2_O-4′), 5.69 (1H, d, *J* = 2.8 Hz, H-1′′), 4.38 (1H, d, *J* = 7.4 Hz, H-1′′′), 4.26 (1H, d, *J* = 7.2 Hz, H-1′′′′). ^13^C-NMR (DMSO-*d*_6_, 100 MHz) δ: 144.7 (C-1), 119.4 (C-2), 128.7 (C-3), 130.3 (C-4), 105.5 (C-5), 150.4 (C-6), 152.5 (C-7), 101.3 (C-8), 129.7 (C-9), 126.4 (C-10), 169.4 (C-11), 67.4 (C-12), 128.6 (C-1′), 109.7 (C-2′), 147.3 (C-3′), 147.7 (C-4′), 108.0 (C-5′), 124.7 (C-6′), 55.8 (OCH_3_-6), 55.2 (OCH_3_-7), 101.2 (3′-OCH_2_O-4′), 109.2 (C-1′′), 104.8 (C-1′′′), 104.2 (C-1′′′′). Compared with the previous reference [28], compound **a** corresponded to procumbenoside E.

Compound **b** (peak **b** in Figure 3B): Positive ESI-MS (*m*/*z*): 543.0 [M + H]^+^. ^1^H-NMR (DMSO-*d*_6_, 400 MHz) δ: 6.94 (1H, d, *J* = 2.0 Hz, H-2′), 7.03 (1H, d, *J* = 8.0 Hz, H-5′), 6.77 (1H, dd, *J* = 8.0, 2.0 Hz, H-6′), 7.00 (1H, s, H-5), 7.68 (1H, s, H-8), 3.68 (3H, s, OCH_3_-6), 3.97 (3H, s, OCH_3_-7), 6.13 (2H, s, 3′-OCH_2_O-4′), 5.50 (1H, d, *J* = 1.5 Hz, H-1′′), 4.40 (1H, d, *J* = 2.8 Hz, H-2′′), 3.78 (1H, d, *J* = 2.8 Hz, H-4′′), 3.49 (1H, s, H-5′′). ^13^C-NMR (DMSO-*d*_6_,100 MHz) δ: 144.6 (C-1), 128.3 (C-2), 126.5 (C-3), 134.3 (C-4), 108.0 (C-5), 150.0 (C-6), 151.8 (C-7), 101.0 (C-8), 128.1 (C-9), 134.4 (C-10), 67.3 (C-11), 169.6 (C-12), 130.8 (C-1′), 111.5 (C-2′), 147.5 (C-3′), 147.5 (C-4′), 106.2 (C-5′), 124.5 (C-6′), 56.1 (OCH_3_-6), 55.8 (OCH_3_-7), 101.6 (3′-OCH_2_O-4′), 110.9 (C-1′′), 76.0 (C-2′′), 78.9 (C-2′′), 75.6 (C-4′′), 66.9 (C-5′′). Compared with the previous reference [29], compound **b** corresponded to diphyllin-1-*O*-β-d-apiofuranoside.

Compound **c** (peak **c** in Figure 3B): Positive ESI-MS (*m*/*z*): 381.0 [M + H]^+^. ^1^H-NMR (DMSO-*d*_6_, 400 MHz) δ: 6.68 (1H, d, *J* = 1.4 Hz, H-2′), 6.85 (1H, d, *J* = 8.0 Hz, H-5′), 6.55 (1H, dd, *J* = 8.0, 1.4 Hz, H-6′), 7.51 (1H, s, H-5), 7.03 (1H, s, H-8), 5.69 (2H, s, H-12), 3.65 (3H, s, OCH_3_-6), 3.95 (3H, s, OCH_3_-7), 9.03 (1H, s, OH-4). ^13^C-NMR (DMSO-*d*_6_, 100 MHz) δ: 130.1 (C-1), 119.1 (C-2), 118.3 (C-3), 147.2 (C-4), 101.2 (C-5), 151.2 (C-6), 150.9 (C-7), 106.5 (C-8), 129.9 (C-9), 124.2 (C-10), 169.1 (C-11), 67.2 (C-12), 125.4 (C-1′), 115.9 (C-2′), 145.1 (C-3′), 145.3 (C-4′), 107.3 (C-5′), 121.7 (C-6′), 55.6 (OCH_3_-6), 56.0 (OCH_3_-7), 101.5 (3′-OCH_2_O-4′). Compared with the previous reference [30], compound **c** corresponded to diphyllin.

Compound **d** (peak **d** in Figure 3B): Positive ESI-MS (*m*/*z*): 381.1 [M + H]^+^. ^1^H-NMR (DMSO-*d*_6_, 400 MHz) δ: 6.71 (1H, s, H-2′), 6.61 (1H, s, H-5′), 7.89 (1H, s, H-4), 6.97 (1H, s, H-5), 7.47 (1H, s, H-8), 5.41 (2H, s, H-12), 9.01 (1H, s, OH-6′), 3.94 (3H, s, OCH_3_-6), 3.68 (3H, s, OCH_3_-7), 6.02 (2H, s, 3′-OCH_2_O-4′). ^13^C-NMR (DMSO-*d*_6_, 100 MHz) δ: 118.9 (C-1), 140.6 (C-2), 119.8 (C-3), 136.4 (C-4), 105.5 (C-5), 151.7 (C-6), 150.3 (C-7), 107.4 (C-8), 128.8 (C-9), 133.0 (C-10), 68.4 (C-11), 167.9 (C-12), 113.1 (C-1′), 110.3 (C-2′), 140.2 (C-3′), 148.5 (C-4′), 98.8 (C-5′), 149.5 (C-6′), 56.7 (OCH_3_-6), 55.2 (OCH_3_-7), 101.9 (3′-OCH_2_O-4′). Compared with the previous reference [31], compound **d** corresponded to 6′-hydroxy justicidin B.

Compounds **e** (peak **e** in Figure 3B): Positive ESI-MS (*m*/*z*): 553.0 [M + H]^+^. ^1^H-NMR (DMSO-*d*_6_, 400 MHz) δ: 6.77 (1 H, d, *J* = 2 Hz, H-2′), 6.96 (1H ,d, *J* = 8 Hz, H-5′), 6.80 (1H, dd, *J* = 8.0, 2.0 Hz, H-6′), 7.60 (1 H, s, H-5), 7.03 (1H, s, H-8), 4.01 (3H, s, OCH_3_-6), 3.96 (3H, s, OCH_3_-7), 6.06 (2H, s, 3′-OCH_2_O-4′), 5.49 (1H, s, H -l”), 4.43 (1H, s, H -2”), 4.05 (1 H, **d**, *J* = 10.1 Hz, H-4”). ^13^C-NMR (DMSO-*d*_6_, 100 MHz) δ: 137.2 (C-1), 118.3 (C-2), 129.0 (C-3), 144.8 (C-4), 100.6 (C-5), 152.1 (C-6 ), 150.5 (C-7), 105.5 (C-8 ), 129.9 (C-9), 126.7 (C-10), 171.2 (C-11), 65.4 (C-12), 129.3 (C-1′), 110.8 (C-2′), 147.7 (C-3′), 148.1 (C-4′), 107.8 (C-5′), 123.7 (C-6′), 56.3 (OCH_3_-6), 56.5 (OCH_3_-7), 101.6 (3′-OCH_2_O-4′), 111.2 (C-1”),78.5 (C-2”), 77.92 (C-3”), 74.9 (C-4”), 66.6 (C-5”). Compared with the previous reference [32], compound **e** corresponded to diphyllin acetyl apioside.

Compound **f** (peak **f** in Figure 3A): Positive ESI-MS (*m*/*z*): 365.1 [M + H]^+^. ^1^H-NMR (CDCl_3_-*d*_1_, 400 MHz) δ: 6.82 (1H, d, *J* = 1.7 Hz, H-2′), 6.95 (1H, d, *J* = 7.9 Hz, H-5′), 6.81 (1H, dd, *J* = 1.7, 7.9 Hz, H-6′), 7.71 (1H, s, H-4), 7.23 (1H, s, H-5), 7.11 (1H, s, H-8), 5.32 (2H, s, H-12) 4.17 (3H, s, OCH_3_-6), 3.87 (3H, s, OCH_3_-7), 6.04 (1H, d, *J* = 1.2 Hz, OCH_2_O-3′), 6.11 (1H, d, *J* = 1.2 Hz, OCH_2_O-4′). ^13^C-NMR (CDCl_3_-*d*_1_, 100 MHz) δ: 139.6 (C-1), 118.5 (C-2), 138.5(C-3), 118.3 (C-4), 106.0(C-5), 151.0(C-6), 152.2(C-7), 108.0(C-8), 128.9 (C-9), 133.4 (C-10), 169.9 (C-11), 68.0 (C-12), 128.4 (C-1′), 110.5 (C-2′), 147.6 (C-3′), 147.5 (C-4′), 106.2 (C-5′), 123.5(C-6′), 56.1 (OCH_3_-6), 55.8 (OCH_3_-7), 101.3 (3′-OCH_2_O-4′). Compared with the previous reference [28], compound **f** corresponded to justicidin B.

Compound **g** (peak **g** in Figure 3A): Positive ESI-MS (*m*/*z*): 395.1 [M + H]^+^. ^1^H-NMR (CDCl_3_-*d*_1_, 400 MHz) δ: 6.93 (1H, d, *J* = 1.3 Hz, H-2′), 6.97 (1H, d, *J* = 7.9 Hz, H-5′), 6.85 (1H, dd, *J* = 1.3, 7.8 Hz, H-6′), 7.52 (1H, s, H-5), 7.03 (1H, s, H-8), 5.65 (2H, s, H-12), 4.15 (3H, s, OCH_3_-4), 4.16 (3H, s, OCH_3_-6), 3.81 (3H, s, OCH_3_-7), 6.07 (1H, d, *J* = 1.3 Hz, OCH_2_O-3′), 6.10 (1H, d, *J* = 1.3 Hz, OCH_2_O-4′). ^13^C-NMR (CDCl_3_-*d*_1_, 100 MHz) δ: 128.5 (C-1), 119.2 (C-2), 130.4 (C-3), 147.7 (C-4), 100.6 (C-5), 150.9 (C-6), 150.1 (C-7), 106.2 (C-8), 134.4 (C-9), 123.6 (C-10), 169.6 (C-11), 66.6 (C-12), 126.5 (C-1′), 110.7 (C-2′), 147.4 (C-3′), 147.8 (C-4′), 108.2 (C-5′), 123.6 (C-6′), 56.6 (OCH_3_-1), 56.1 (OCH_3_-6), 55.8 (OCH_3_-7), 101.2 (3′-OCH_2_O-4′). Compared with the previous reference [31], compound **g** corresponded to justicidin A.

Compound **h** (peak **h** in Figure 3A): Positive ESI-MS (*m*/*z*): 411.1 [M + H]^+^. ^1^H-NMR (CDCl_3_-*d*_1_, 400 MHz) δ: 6.93 (1H, s, H-2′), 7.06 (1H, s, H-5′), 6.95 (1H, s, OH-6′), 7.73 (1H, s, H-5), 6.85 (1H, s, H-8), 5.15 (2H, s, H-12), 4.48 (3H, s, OCH_3_-4), 4.12 (3H, s, OCH_3_-6), 3.93 (3H, s, OCH_3_-7), 6.06 (1H, d, *J* = 1.3 Hz, OCH_2_O-3′), 6.11 (1H, d, *J* = 1.3 Hz, OCH_2_O-4′). ^13^C-NMR (CDCl_3_-*d*_1_, 100 MHz) δ: 129.3 (C-1), 109.2 (C-2), 133.4 (C-3), 148.7 (C-4), 101.5 (C-5), 155.5 (C-6), 152.2 (C-7), 104.0 (C-8), 139.9 (C-9), 123.6 (C-10), 169.6 (C-11), 68.6 (C-12), 126.5 (C-1′), 109.7 (C-2′), 147.4 (C-3′), 149.4 (C-4′), 109.2 (C-5′), 123.76 (C-6′), 56.1 (OCH_3_-6), 55.9 (OCH_3_-7), 102.2 (3′-OCH_2_O-4′). Compared with the previous reference [33], compound **h** corresponded to 6′-hydroxyjusticidin C.

Compound **i** (peak **i** in Figure 3A): Positive ESI-MS (*m*/*z*): 349.1 [M + H]^+^. ^1^H-NMR (CDCl_3_-*d*_1_, 400 MHz) δ: 6.83 (1H, d, *J* = 1.7 Hz, H-2′), 6.87 (1H, d, *J* = 8.0 Hz ,H-5′), 6.68 (1H, dd, *J* = 1.7, 8.0 Hz, H-6′), 8.27 (1H, s, H-1), 7.28 (1H, s, H-5), 7.33 (1H, s, H-8); ^13^C-NMR (CDCl_3_-*d*_1_, 100 MHz) δ: 124.7 (C-1), 137.4 (C-2), 120.7 (C-3), 131.3(C-4), 101.5 (C-5), 147.4 (C-6), 151.5 (C-7), 105.3 (C-8), 133.1 (C-9), 133.4 (C-10), 172.4 (C-11), 69.4 (C-12), 129.6 (C-1′), 109.7 (C-2′), 147.3 (C-3′), 148.7 (C-4′), 109.0 (C-5′), 122.7 (C-6′), 102.2 (6-OCH_2_O-7), 101.7 (3′-OCH_2_O-4′). Compared with the previous reference [13], compound **i** corresponded to justicidin E. 

Compound **j** (peak **j** in Figure 3A): Positive ESI-MS (*m*/*z*): 379.0 [M + H]^+^. ^1^H-NMR (CDCl_3_-*d*_1_, 400 MHz) δ: 6.77 (1H, d, H-2′), 6.86 (1H, d, H-5′), 6.99 (1H, m, H-6′), 7.81 (1H, s, H-5), 7.04 (1H, s, H-8), 5.20 (2H, s, H-12), 4.35 (3H, s, OCH_3_-4), 6.09 (2H, dd, *J* = 4.5 Hz, 6-OCH_2_O-7), 6.11 (2H, dd, *J* = 4.6, 3.7 Hz, 3′-OCH_2_O-4′). ^13^C-NMR (CDCl_3_-*d*_1_, 100 MHz) δ: 129.7 (C-1), 139.4 (C-2), 122.7 (C-3), 150.3 (C-4), 121.5 (C-5), 138.4 (C-6), 138.5 (C-7), 100.3 (C-8), 135.7 (C-9), 127.4 (C-10), 169.4 (C-11), 68.4 (C-12), 129.6 (C-1′), 109.7 (C-2′), 148.3 (C-3′), 147.8 (C-4′), 109.0 (C-5′), 125.7 (C-6′), 101.8 (3′-OCH_2_O-4′), 101.4 (6-OCH_2_O-7). Compared with the previous reference [33], compound **j** corresponded to lignan J_1_.

## 4. Conclusions

The satisfactory isolation strategy of 10 lignans from a crude extract of *J. procumbens* was successfully optimized and established by HSCCC. From 320 mg of the crude sample, 10 lignans were obtained, including procumbenoside E (12.1 mg), diphyllin-1-*O*-β-d-apiofuranoside (7.6 mg), diphyllin (7.4 mg), 6′-hydroxy justicidin B (8.3 mg), diphyllin acetyl apioside (7.9 mg), justicidin B (19.3 mg), justicidin A (10.8 mg), 6′-hydroxyjusticidin C (13.9 mg), justicidin E (7.7 mg), and lignan J_1_ (6.3 mg) with purity all over 94.0%. The study is of great reference value for obtaining high purity lignans and their glycosides from *J. procumbens*, which also indicated that HSCCC is a powerful technique for the separation and purification of active compounds with a wide range of polarity from natural products.

## Figures and Tables

**Table 1:** The *K*_D_ values of target compounds in a series of solvent systems.

Sample	Solvent System (*v*/*v*): (Pet–EtOAc–MeOH–H_2_O)	Partition Coefficient (*K*_D_)									
		a	b	c	d	e	f	g	h	i	j
Crude sample	3:3:3:3	0.12	0.25	0.34	0.81	1.28	4.06	6.18	7.12	7.89	12.10
	3:2.4:3:2.4	0.09	0.19	0.29	0.68	1.06	2.52	4.33	5.41	5.97	6.43
	3:2.1:3:2.1	0.07	0.11	0.18	0.23	0.42	0.74	1.35	1.84	2.18	2.44
	3:1.8:3:1.8	0.05	0.08	0.10	0.16	0.31	0.37	0.67	1.26	1.54	2.07
Enriched mixture	3:3.2:3:3.2	0.17	0.30	0.44	0.93	1.44					
	3:3.8:3:3.8	0.22	0.68	0.93	1.75	2.08					
	3:4.2:3:4.2	0.35	1.34	1.86	2.52	4.03					
